# A Survey on the Effect of Livestock Production System and Finishing Diet on Sensory Characteristics of Foal Meat Using Generalized Procrustes Analysis

**DOI:** 10.1155/2016/8729053

**Published:** 2016-02-23

**Authors:** José M. Lorenzo, Laura Purriños, Javier Carballo

**Affiliations:** ^1^Centro Tecnológico de la Carne de Galicia, Rúa Galicia No. 4, Parque Tecnológico de Galicia, San Cibrán das Viñas, 32900 Ourense, Spain; ^2^Area de Tecnología de los Alimentos, Facultad de Ciencias de Ourense, Universidad de Vigo, 32004 Ourense, Spain

## Abstract

The influence of livestock production system [Freedom Extensive System (FES) versus Semi-Extensive System (SES)] and finishing feed (1.5 kg versus 3.0 kg of commercial feed in the finishing period) diet on sensory properties of foal meat using Generalized Procrustes Analysis (GPA) was studied. For this work, a total of 24 foals (8 from FES and 16 from SES) were used. Samples were evaluated by eight panelists for eight sensory attributes: colour, marbling, odour intensity, sweetness, springiness, hardness, chewiness, and juiciness. Data were analyzed using a GPA to minimize differences among testers. Highly appreciated sensory properties (odour intensity, red colour, marbling, and juiciness) were mostly associated with foals from the Semi-Extensive System. On the other hand, the three groups studied (FES, 1.5SES, and 3SES) were clearly recognized by panelists on the consensus configuration and they were clearly separated on the map. This study concluded that sensory characteristics of foal meat from a Semi-Extensive Production System with a finishing diet of 3 kg concentrate were more preferable than the other ones.

## 1. Introduction

Horse meat production is important in the EU (140 698 Mt in 2013), Italy being the first producer within the EU in 2013 with 15,179 Mt, followed by Poland (12,000), Spain (11,668), and Romania (9,180) [1]. Despite the fact that horse meat consumption increased in recent years, it is not comparable to the consumption that occurs with other types of meats such as beef, chicken, or pork, which are more important in the human diet [2]. This increase might be due to changes in attitude towards this type of meat and the interest of the consumers in tasting new meat products [3].

This meat presents positive characteristics from a nutritional point of view, and in line with recent health recommendations concerning fatty acids and lipids consumption, being regarded as a “dietary” meat. Equine meat is characterized by low fat [4], low cholesterol content [5], richness in Fe-heme [6], and high level of unsaturated fatty acids (above 55%) [3, 5, 7, 8].

The sensory characteristics of meat remain one of the main factors influencing consumers' satisfaction, since sensory properties like colour or tenderness may have a significant impact on eating quality and general acceptability. Sensory analysis performed by trained panelists is the most appropriate tool to explain differences between the treatments as perceived by humans [9].

Livestock production system, including management and feeding, has a great effect on the meat characteristics of the monogastric animals. However, studies on this topic in equines are very scarce, and there are no studies evaluating the sensorial characteristics of horse meat from different livestock production systems.

Generalized Procrustes Analysis (GPA) [10] is a powerful multivariate technique extensively used in sensory evaluation. The analysis minimizes differences between assessors, identifies agreement between them, and summarizes the sets of 3-dimensional data (objects, characteristics, and assessors). In GPA the data matrices of individual panelists are subjected to rotation and, optionally, translation and stretching/shrinking to maximize the agreement among the testers. It has the advantage of being a multivariate method, thus dealing with all descriptors and all testers at one. GPA calculates the consensus configuration of the sample and enables us to present graphical results in a two-dimensional map.

The objective of this work was to study the effect of livestock production system and finishing diet on sensory properties of foal meat using Generalized Procrustes Analysis, in order to generate information that helps to improve the production systems for offering horse meat of higher quality in the markets.

## 2. Materials and Methods

### 2.1. Experimental Design and Animal Management

For this study, twenty-four foals from crossing Galician Mountain × Hispano-Bretón (GM × HB) were used.

Eight foals were obtained from Monte Cabalar (agricultural cooperative of “Galician Mountain”) located in a mountainous region (A Estrada, Pontevedra, Spain). Animals were reared with their mothers on pasture and they were kept suckling and grazing until the weaning age at 6-7 months. After weaning, foals were fed mainly with ryegrass* (Lolium perenne*),* Ulex europaeus *L., and* Pteridium aquilinum *(L.) Kuhn, receiving complementary grass silage* ad libitum* when the grass available was limited, especially in the summer and winter time. All foals were reared with their mothers in an extensive production system in freedom regimen, according to an extensive production system on wood pasture. Animals that belong to this herd were denominated as Freedom Extensive System (FES).

The other sixteen foals were obtained from an experimental herd of the Agricultural Research Centre of Mabegondo (Marco da Curra, A Coruña, Spain). Animals were reared with their mothers on pasture and were allowed to suckle freely until 6–8 months old. Next, they were fed with commercial feeding and pasture for 4 months (from May to September, the months where the pasture has the best conditions of amount and quality). At this point, animals were randomly divided into two groups that were fed with two different amounts of concentrate [*n* = 8: 1.5 kg of fodder/foal-day (denominated 1.5SES) and *n* = 8: 3.0 kg of fodder/foal-day (denominated 3SES)]. Commercial feed was composed of barley, corn, soybean flour, wheat bran, alfalfa, sugar cane molasses, beet, animal fat, calcium carbonate, sodium chloride, and powder lactose; its proximate composition (%) was crude protein (15.1), crude fibre (6.7), ashes (5.5), fat (4.5), and sodium (0.2). This ration was supplemented with a mineral/vitamin mix having the following composition: vitamin A (6000 IU/kg); vitamin D3 (600 IU/kg); minerals expressed in mg/kg: zinc (150), manganese (70), iron (90), cooper (10), cobalt (0.30), and iodine (2); butyl-hydroxyanisole (0.03 mg/kg); ethoxyquin (0.03 mg/kg). There was a period of adaptation to the commercial feeding, in order to avoid colic syndromes, which usually appear with a sudden change in the diet. The amount of commercial feed was gradually increased, starting with small quantities to reach the final amount. The period of adaptation was 20 and 30 days for each group (1.5SES and 3SES, resp.). Animals that belong to this herd, being managed in a Semi-Extensive System, were described as Semi-Extensive Production System (SES).

All foals were slaughtered at the age of eighteen months. They were transported to the abattoir (distance around 70 km and 15 km, for SES and FES, resp.) the day before slaughter, without mixing foals with different groups at any time, trying to minimize the stress of the animals. The animals were stunned with a captive bolt and slaughtered and dressed according to the specifications outlined in the European legislation (Council Directive 93/119/EC).

### 2.2. Sample Collection

Immediately after slaughter, carcasses were weighed and chilled at 4°C in a cold chamber for 24 h. After 7 days of storage, the* longissimus dorsi* (LD) muscle was taken from the left half of each carcass between the fifth and the tenth ribs. Samples were transported immediately to the laboratory under refrigerated conditions (<4°C). Samples were packed under vacuum conditions (96%) (FRIMAQ V-900 device, Lorca, Murcia, Spain) and stored at −20°C until evaluation.

### 2.3. Sensory Analysis

The evaluation was conducted by eight panelists that were selected and trained in accordance with the ISO 8586:2012 standard [11]. Panel members were situated in a private cabinet during sessions. Water to clean the palate and remove residual flavours was given to them at the beginning of the performance and between samples. Panelists were firstly trained during 5 sessions, evaluating, describing, and discussing the foal meat quality properties which were later evaluated.

Sensory attributes evaluated were colour and marbling in fresh meat and odour intensity, sweetness, springiness, hardness, chewiness, and juiciness in cooked meat.

The sensory evaluation consisted of two steps: first, visual evaluation of fresh meat (colour and marbling) and, second, assessment of all sensory attributes after cooking. Samples were individually labelled with three-digit random numbers and were randomly served. The sensory evaluation consisted of eight sessions. In each sensory session, panelist evaluated 3 samples using a 10 cm unstructured line, representing at the extremes the minimum (minimum intensity) and the maximum (maximum intensity). Panelists were asked to indicate a point on the scale corresponding to the intensity of their different feelings for each attribute, and then each one was measured using a 10 cm ruler to score it from 0 to 10 points. The tasting order was designed to avoid first sample and carryover effects [12].

To colour evaluation, meat samples (25 mm thick) were exposed to air for 30 min at 4°C to allow complete bloom, prior to evaluation.

The samples for the cooked meat evaluation were cut into 25 mm thick segments. Steaks were cooked in an oven (Siemens mod. HB370560E, Madrid, Spain) at 200°C, inside aluminum paper, until they reached 70°C of internal temperature, which was monitored by an internal thermocouple (HI-985011, Hanna Instruments, Spain). The cooked steaks were cut into 10 × 10 × 25 mm^3^ pieces and wrapped in codified aluminum foil and stored in a warm cabinet at 50°C until tasting.

### 2.4. Statistical Analysis

For the statistical analysis of the results, firstly an analysis of variance (ANOVA) of one way using the IBM SPSS Statistics 19.0 (IBM Corporation, Somers, NY, USA) was performed for all variables considered in the study. The least squares mean (LSM) was separated using Duncan's *t*-test. All statistical tests of LSM were performed for a significance level *P* < 0.05. Correlations between variables were established by correlation analyses using Pearson's linear correlation coefficient with the above-mentioned statistical software package.

For the Generalized Procrustes Analysis (GPA), some graphical displays of the results were used. The data matrices of 24 (foal meat samples) by 8 (sensory attributes) for the 8 assessors (configurations) were matched to find a consensus using the Microsoft Office Excel add-in software, XLSTAT (Version 4.02, Addinsoft, Paris, France).

## 3. Results and Discussion

Mean scores and standard deviations for sensory characteristics of the three groups studied are shown in Table 1. No significant differences (*P* > 0.05) were observed among the three groups studied. Results obtained from Pearson correlation test indicated that only three attributes showed significantly positive correlations (Table 2). As can be seen, positive correlation between sweetness and marbling (*r* = 0.556; *P* < 0.01), springiness and odour intensity (*r* = 0.496; *P* < 0.05), and juiciness and sweetness (*r* = 0.456; *P* < 0.05) was found. Since no significant (*P* > 0.05) sensory differences were found among the three groups (see Table 1), the use of a multivariate analysis is a valuable tool for reducing collinearity among attributes.

Panelists profiled eight attributes to describe the differences among the three groups studied.  Table 3 shows the residual variance by the three groups studied after the transformations. As can be seen, the 3SES group presented the lowest residual variance, which indicates that there was most probably more coincidence among experts compared with the other two groups.

The results obtained for the residual variance, scaling factors, and the percentage variation accounted for are shown in Table 4. Five out of 8 assessors had a low residual variance (<1%), indicating homogeneity within the group. Each evaluator's residual variance reveals the relationship between the individual sample map and the consensus sample map (produced by the data obtained from the 8 evaluators). The residual variance of assessors provides a measurement of goodness of fit of the consensus space of each assessor. It is expected that this can be as small as possible in order to facilitate a greater adjustment. As can be seen, assessors 2 and 7 had the highest residual variance. On the other hand, panelists 1, 3, 5, 6, and 8 tend to use a wider part of the scale, since their scaling factors were higher than 1, while testers 2, 4, and 7 used a narrower part of the scale, since their scaling factors were lower than 1. In any case, and as Alcalde et al. [13] pointed out, the low levels of residual variance from the panelists in comparison to the values reported in other studies [9, 14] and the homogeneity of the scaling values observed in the present study indicated a reasonable efficacy in the training of the panel used in the evaluation of the foal meat.

**Figure 1:**
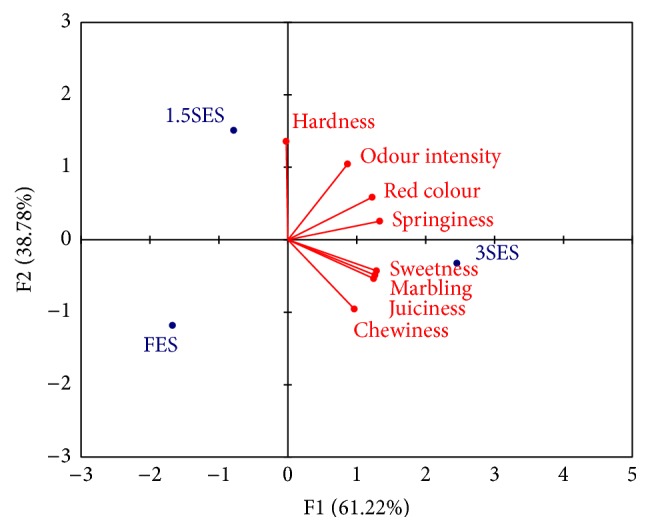
Consensus configuration: joint representation of the correlation between the sensory parameters and their first two dimensions, and groups of animal's meat coordinates for foal sensory analysis. F1: first principal component of Generalized Procrustes Analysis (GPA); F2: second principal component of GPA; FES: Freedom Extensive System; 1.5SES: Semi-Extensive System with 1.5 kg of fodder/foal-day; and 3SES: Semi-Extensive System with 3 kg of fodder/foal-day.

To minimize the differences among panelists, a GPA was used to find a coincidence (Figure 1); GPA correct the different use of the scale by panelists and this even allows using semitrained panelists. A great degree of coincidence was found between the eight testers with regard to the configuration of the three groups (FES, 1.5SES, and 3SES). After optimization, the first two main axes of the consensus explained 100% of the total variation among groups, since 61.2% of consensus variance was accounted for for the first dimension, while the second dimension explained 38.8% of the variance.

Eigenvalues and correlation between sensory parameters and GPA factors (F1 and F2) are shown in Table 5. According to Table 5, the highest eigenvalue (1.77) was observed for the first factor. On the other hand, red colour, marbling, sweetness, springiness, chewiness, and juiciness were high and positively correlated with F1 (Table 5), while hardness and odour intensity were high and positively correlated with F2, and chewiness negatively correlated with F2. Thus, it can be stated that almost all the sensory properties play an important role in formation of the first factor.

Figure 1 also displays the coordinate of three groups, after the principal component analysis, and the correlation between sensory parameters and the first two dimensions. FES and 1.5SES groups were classified in the same region for dimension 1, while 3SES group was placed in the positive part of F1. This finding shows that the panelists distinguished foal meat from 3SES group to the other ones. When the second dimension was taken into consideration, 1.5SES group was located in the positive region, while the other ones were located in the negative region.

The sensory properties odour intensity, red colour, and springiness were located in the positive axis of F1 and F2; hardness was located in the negative axis of F1 and in the positive axis of F2, and sweetness, juiciness, marbling, and chewiness were located in the positive region of F1 and in the negative region of F2. In Figure 1, we can see that almost all attributes are in the positive F1. This indicates that in this region meat has higher scores for these attributes; at the same time, it indicates that in the opposite direction meat has lower scores for these attributes. The 3SES group was found highly correlated with the sensory characteristics for the first dimension, and this group may be considered as negatively correlated with the sensory attributes of the second dimension. The 1.5SES group was negatively correlated with the sensory characteristics of F1 and positively correlated with the sensory attributes of F2. Finally, the FES group was negatively correlated with all the attributes, both the F1 and F2.

Foal meat from Semi-Extensive System with 3 kg of fodder/foal-day seems to be the most pleasant for consumers. Risvik [15] noticed that the consumers generally prefer tender and juicy meat. Highly appreciated sensory properties (odour intensity, red colour, marbling, and juiciness) were mostly associated with semi-extensively reared animals. Thus, a meat production system with a finishing diet of 3 kg of fodder/foal-day may be expected to produce foal meat that the consumers would prefer. Although the 1.5SES group was located in a different axis for the second dimension, it can be stated that the 1.5SES group was also moderately related to sensory characteristics, and finishing diet of 1.5 kg of fodder/foal-day may be also acceptable.

Colour is one of the most critical characteristics that the consumers consider when making a decision to purchase meat [16] and it is a visual parameter associated with the freshness [17]. Our study showed that colour was related to the 3SES group, which is in agreement with results reported by Resconi et al. [18], who found that the use of concentrate or grass silage influenced colour, odour, and flavour intensity of beef. On the other hand, Franco et al. [7] investigated the relationships between finishing diet and some concrete sensory characteristics in foal meat and pointed out that the foals with higher amount of concentrate displayed greater juiciness; this fact could be related with the greater intramuscular fat content as reported in other works carried out on pork meat [14].

**Figure 2:**
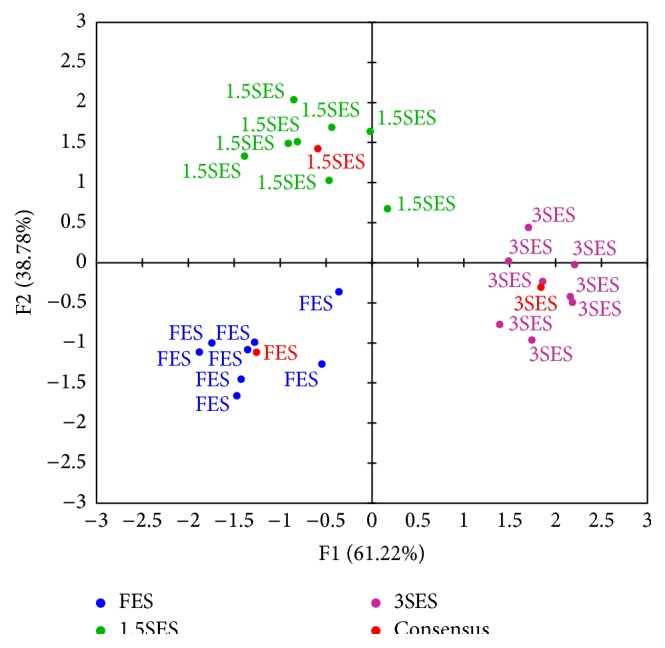
Consensus configuration by object (foal groups: FES, 1.5SES, and 3SES) from the sensorial analysis. F1: first principal component of Generalized Procrustes Analysis (GPA); F2: second principal component of GPA; FES: Freedom Extensive System; 1.5SES: Semi-Extensive System with 1.5 kg of fodder/foal-day; 3SES: Semi-Extensive System with 3 kg of fodder/foal-day.

A map of the different types of meat grouped by livestock production system and finishing diet is shown in Figure 2. The points are close to the first axis as a result of 61.2% of the variability concentrated on this axis. The three groups (FES, 1.5SES, and 3SES) were clearly recognized by panelists on the consensus configuration and the three groups were clearly separated on the map. As can be seen, mean values for 3SES group were at the positive side of F1 and in the negative side of F2, 1.5SES group appeared at F1 < 0 and in the positive side of F2, and FES group was at the negative side of F1 and F2.

In line with our results, GPA method was satisfactorily used in previous works to assess the effect of sex, genotype (breed), or carcass weight on sensory characteristics of meat from lambs [13, 19], pork [14], and goats [9, 20]. As occurred in these studies carried out on meat from other animal species, GPA probed to be a good method to analyze the sensory characteristics of foal meat and also to know how individual judges differ or agree in their perceptions on the same meat sample.

## 4. Conclusions

Semi-Extensive System with 3 kg of concentrate improved the organoleptic quality of the meat, mainly by increasing springiness, sweetness, marbling, and juiciness and reducing its hardness. These animals also had the highest red colour and the lowest intensity of odour. From the results obtained in the present study it can be concluded that the production system and finishing feeding had an important effect on the sensory attributes of foal meat. Finally, this study suggests the usefulness of the application of GPA methodology in discriminating foal meat from different production systems and finishing diets.

## Figures and Tables

**Table 1:** Values of sensory attributes for the three foal groups (means ± standard deviations of eight qualifications).

	FES	1.5SES	3SES	SEM	*P* value
Red colour	4.36 ± 0.47	4.71 ± 0.74	4.88 ± 0.56	0.12	0.233
Marbling	3.46 ± 0.95	3.35 ± 1.10	4.06 ± 0.74	0.19	0.291
Odour intensity	4.37 ± 1.08	4.53 ± 0.64	4.52 ± 1.06	0.18	0.931
Sweetness	3.62 ± 1.24	3.52 ± 1.11	4.42 ± 1.53	0.26	0.341
Springiness	3.90 ± 0.68	4.32 ± 1.31	5.01 ± 1.08	0.22	0.131
Hardness	3.82 ± 0.94	4.42 ± 0.62	4.00 ± 0.50	0.14	0.251
Chewiness	4.31 ± 0.41	4.00 ± 1.18	4.58 ± 1.18	0.20	0.510
Juiciness	4.27 ± 0.74	4.12 ± 0.62	4.81 ± 0.49	0.13	0.094

FES: Freedom Extensive System; 1.5SES: Semi-Extensive System with 1.5 kg of fodder/foal-day; 3SES: Semi-Extensive System with 3 kg of fodder/foal-day.

SEM: standard error of mean.

**Table 2:** Correlation coefficients among sensory attributes.

	Red colour	Marbling	Odour intensity	Sweetness	Springiness	Hardness	Chewiness	Juiciness
Red colour	1							
Marbling	−0.338	1						
Odour intensity	0.378	0.000	1					
Sweetness	0.009	0.556^*∗∗*^	0.037	1				
Springiness	0.288	0.211	0.496^*∗*^	0.146	1			
Hardness	0.212	0.031	0.133	0.160	−0.028	1		
Chewiness	0.122	0.142	0.000	0.377	0.239	0.080	1	
Juiciness	0.205	0.302	0.050	0.456^*∗*^	0.186	−0.275	0.091	1

Significance: ^*∗*^ *P* < 0.05, ^*∗∗*^ *P* < 0.01.

**Table 3:** Residual variance for each foal group.

Animal group	Residual
FES	3.046
1.5SES	3.025
3SES	2.161

FES: Freedom Extensive System; 1.5SES: Semi-Extensive System with 1.5 kg of fodder/foal-day; 3SES: Semi-Extensive System with 3 kg of fodder/foal-day.

**Table 4:** Residual variance, scaling factors, and percentage variation explained by the first two principal components for each tester for foal sensory analysis.

Tester	Residual	Scaling factor	F1^a^%	F2^b^%
1	0.636	1.570	79.808	20.192
2	2.462	0.630	64.193	35.807
3	0.944	1.272	44.592	55.408
4	1.385	0.900	31.845	68.155
5	0.181	1.198	66.276	33.724
6	0.276	1.085	67.707	32.293
7	2.105	0.831	77.369	22.631
8	0.242	1.200	53.041	46.959

^a^F1: first principal component of Generalized Procrustes Analysis (GPA).

^b^F2: second principal component of GPA.

**Table 5:** Eigenvalues and correlation between the sensory attributes and the two principal components (F1 and F2) of the Generalized Procrustes Analysis (GPA).

	Eigenvalues		Correlations between sensory attributes and factor		
	F1	F2		F1	F2
Eigenvalue	1.77	1.12	Red colour	0.902	0.432
Variability (%)	61.22	38.78	Marbling	0.933	−0.360
Cumulative (%)	61.22	100.00	Odour intensity	0.640	0.769
			Sweetness	0.949	−0.315
			Springiness	0.982	0.189
			Hardness	−0.017	1.000
			Chewiness	0.711	−0.703
			Juiciness	0.919	−0.395
